# Dichlorido(2,4,6-tri-2-pyridyl-1,3,5-triazine)manganese(II)

**DOI:** 10.1107/S1600536810004204

**Published:** 2010-02-06

**Authors:** Kwang Ha

**Affiliations:** a School of Applied Chemical Engineering, The Research Institute of Catalysis, Chonnam National University, Gwangju 500-757, Republic of Korea

## Abstract

In the title complex, [MnCl_2_(C_18_H_12_N_6_)], the Mn^II^ ion is five-coordinated in an approximately square-pyramidal geometry defined by three N atoms of the tridentate 2,4,6-tri-2-pyridyl-1,3,5-triazine ligand and two Cl atoms. In the crystal, the mol­ecules are stacked in columns along the *c* axis and display inter­molecular π–π inter­actions between the six-membered rings, the shortest centroid–centroid distance being 3.553 (3)Å. Inter­molecular C—H⋯Cl contacts are also noted.

## Related literature

For the crystal structure of 2,4,6-tri-2-pyridyl-1,3,5-triazine (tptz), see: Drew *et al.* (1998 ▶). For the crystal structures of some other Mn(II)–tptz complexes, see: Hsu *et al.* (2006 ▶); Majumder *et al.* (2006 ▶); Sun *et al.* (2007 ▶); Tyagi & Singh (2009 ▶); Zhang *et al.* (2008 ▶); Zhao *et al.* (2007 ▶).
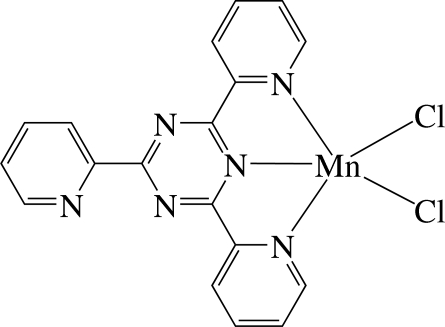


## Experimental

### 

#### Crystal data

[MnCl_2_(C_18_H_12_N_6_)]
*M* *_r_* = 438.18
Triclinic, 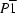

*a* = 8.8247 (7) Å
*b* = 10.5538 (9) Å
*c* = 10.9635 (9) Å
α = 66.572 (2)°
β = 75.812 (2)°
γ = 82.867 (2)°
*V* = 907.91 (13) Å^3^
*Z* = 2
Mo *K*α radiation
μ = 1.04 mm^−1^
*T* = 200 K
0.32 × 0.13 × 0.06 mm

#### Data collection

Bruker SMART 1000 CCD diffractometer
Absorption correction: multi-scan (*SADABS*; Bruker, 2000 ▶) *T*_min_ = 0.856, *T*_max_ = 1.000
6800 measured reflections
4424 independent reflections
2256 reflections with *I* > 2σ(*I*)
*R*_int_ = 0.049

#### Refinement

*R*[*F* ^2^ > 2σ(*F* ^2^)] = 0.061
*wR*(*F* ^2^) = 0.163
*S* = 1.05
4424 reflections
244 parameters
H-atom parameters constrained
Δρ_max_ = 0.73 e Å^−3^
Δρ_min_ = −0.86 e Å^−3^

### 

Data collection: *SMART* (Bruker, 2000 ▶); cell refinement: *SAINT* (Bruker, 2000 ▶); data reduction: *SAINT*; program(s) used to solve structure: *SHELXS97* (Sheldrick, 2008 ▶); program(s) used to refine structure: *SHELXL97* (Sheldrick, 2008 ▶); molecular graphics: *ORTEP-3* (Farrugia, 1997 ▶) and *PLATON* (Spek, 2009 ▶); software used to prepare material for publication: *SHELXL97*.

## Supplementary Material

Crystal structure: contains datablocks global, I. DOI: 10.1107/S1600536810004204/tk2624sup1.cif

Structure factors: contains datablocks I. DOI: 10.1107/S1600536810004204/tk2624Isup2.hkl

Additional supplementary materials:  crystallographic information; 3D view; checkCIF report

## Figures and Tables

**Table 1:** Hydrogen-bond geometry (Å, °)

*D*—H⋯*A*	*D*—H	H⋯*A*	*D*⋯*A*	*D*—H⋯*A*
C10—H10⋯Cl1^i^	0.95	2.77	3.594 (5)	145
C15—H15⋯Cl1^ii^	0.95	2.82	3.714 (5)	157

Symmetry codes: (i) 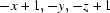; (ii) 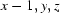.

**Fig. 1.**
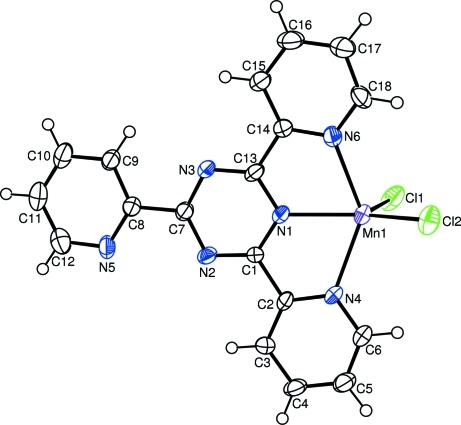
The structure of the title complex, with displacement ellipsoids drawn at the 50% probability level for non-H atoms.

**Fig. 2.**
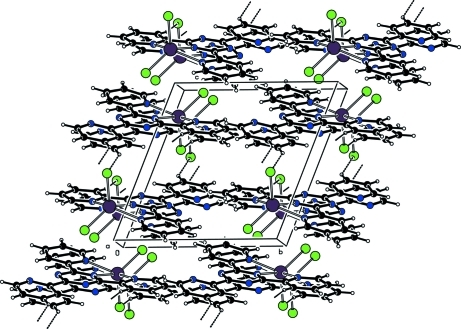
View of the unit-cell contents of the title complex. Hydrogen-bond interactions are drawn with dashed lines.

## supplementary crystallographic information

### Comment

Since the original structure determination of 2,4,6-tri-2-pyridyl-1,3,5-triazine ligand (Drew *et al.*, 1998), triazine complexes, including Mn(II) derivatives, have attracted considerable interest over the years (Hsu *et al.*, 2006; Majumder *et al.*, 2006; Sun *et al.*, 2007; Tyagi & Singh, 2009; Zhang *et al.*, 2008; Zhao *et al.*, 2007). In the title complex, [MnCl_2_(C_18_H_12_N_6_)], the Mn^II^ ion is five-coordinated in an approximately square-pyramidal geometry by three N atoms of the tridentate 2,4,6-tri-2-pyridyl-1,3,5-triazine ligand and two Cl atoms (Fig. 1). While the Mn—Cl bond lengths are almost equal [2.3345 (16) and 2.3494 (16) Å], the Mn—N bond lengths appear to be different (Table 1). The Mn—N(pyridyl) bonds [2.303 (4) and 2.324 (4) Å] tend to be slightly longer than the Mn—N(triazine) bond (2.197 (4) Å]. The N—Mn—N chelating angles are 70.05 (13)° and 70.68 (14)°, and the Cl—Mn—Cl bond angle is 112.22 (6) °. The molecules are stacked in columns along the c axis and display intermolecular π-π interactions between the six-membered rings, with a shortest centroid-centroid distance of 3.553 (3) Å, and weak intermolecular C—H···Cl contacts (Fig. 2 and Table 2).

### Experimental

To a solution of 2,4,6-tri-2-pyridyl-1,3,5-triazine (0.25 g, 0.80 mmol) in EtOH (30 ml) was added MnCl_2_.4H_2_O (0.16 g, 0.81 mmol) and stirred for 2 h at room temperature. The formed precipitate was separated by filtration and washed with EtOH and dried under vacuum, to give a yellow powder (0.14 g). Crystals suitable for X-ray analysis were obtained by slow evaporation from a CH_3_CN solution.

### Refinement

H atoms were positioned geometrically and allowed to ride on their respective parent atoms [C—H = 0.95 Å and U_iso_(H) = 1.2U_eq_(C)].

### Crystal data

**Table:**

[MnCl_2_(C_18_H_12_N_6_)]	*Z* = 2
*M**_r_* = 438.18	*F*(000) = 442
Triclinic, *P*1	*D*_x_ = 1.603 Mg m^−^^3^
Hall symbol: -P 1	Mo *K*α radiation, λ = 0.71073 Å
*a* = 8.8247 (7) Å	Cell parameters from 1442 reflections
*b* = 10.5538 (9) Å	θ = 2.3–24.8°
*c* = 10.9635 (9) Å	µ = 1.04 mm^−^^1^
α = 66.572 (2)°	*T* = 200 K
β = 75.812 (2)°	Plate, yellow
γ = 82.867 (2)°	0.32 × 0.13 × 0.06 mm
*V* = 907.91 (13) Å^3^	

### Data collection

**Table:**

Bruker SMART 1000 CCD diffractometer	4424 independent reflections
Radiation source: fine-focus sealed tube	2256 reflections with *I* > 2σ(*I*)
graphite	*R*_int_ = 0.049
φ and ω scans	θ_max_ = 28.3°, θ_min_ = 2.1°
Absorption correction: multi-scan (*SADABS*; Bruker, 2000)	*h* = −9→11
*T*_min_ = 0.856, *T*_max_ = 1.000	*k* = −14→12
6800 measured reflections	*l* = −14→14

### Refinement

**Table:**

Refinement on *F*^2^	Primary atom site location: structure-invariant direct methods
Least-squares matrix: full	Secondary atom site location: difference Fourier map
*R*[*F*^2^ > 2σ(*F*^2^)] = 0.061	Hydrogen site location: inferred from neighbouring sites
*wR*(*F*^2^) = 0.163	H-atom parameters constrained
*S* = 1.05	*w* = 1/[σ^2^(*F*_o_^2^) + (0.0495*P*)^2^ + 0.2689*P*] where *P* = (*F*_o_^2^ + 2*F*_c_^2^)/3
4424 reflections	(Δ/σ)_max_ < 0.001
244 parameters	Δρ_max_ = 0.73 e Å^−^^3^
0 restraints	Δρ_min_ = −0.86 e Å^−^^3^

### Special details

**Table:**

Geometry. All esds (except the esd in the dihedral angle between two l.s. planes) are estimated using the full covariance matrix. The cell esds are taken into account individually in the estimation of esds in distances, angles and torsion angles; correlations between esds in cell parameters are only used when they are defined by crystal symmetry. An approximate (isotropic) treatment of cell esds is used for estimating esds involving l.s. planes.
Refinement. Refinement of *F*^2^ against ALL reflections. The weighted *R*-factor wR and goodness of fit *S* are based on *F*^2^, conventional *R*-factors *R* are based on *F*, with *F* set to zero for negative *F*^2^. The threshold expression of *F*^2^ > σ(*F*^2^) is used only for calculating *R*-factors(gt) etc. and is not relevant to the choice of reflections for refinement. *R*-factors based on *F*^2^ are statistically about twice as large as those based on *F*, and *R*- factors based on ALL data will be even larger.

### Fractional atomic coordinates and isotropic or equivalent isotropic displacement parameters (Å^2^)

**Table:**

	*x*	*y*	*z*	*U*_iso_*/*U*_eq_	
Mn1	1.11311 (8)	−0.09650 (8)	0.20984 (8)	0.0315 (2)	
Cl1	1.19313 (15)	−0.18383 (16)	0.41816 (14)	0.0489 (4)	
Cl2	1.27174 (15)	−0.17981 (15)	0.05186 (14)	0.0474 (4)	
N1	0.8963 (4)	0.0276 (4)	0.2302 (4)	0.0273 (9)	
N2	0.7690 (4)	0.2386 (4)	0.2287 (4)	0.0275 (9)	
N3	0.6232 (4)	0.0355 (4)	0.2991 (4)	0.0280 (9)	
N4	1.1690 (4)	0.1343 (4)	0.1225 (4)	0.0293 (10)	
N5	0.4828 (5)	0.3769 (4)	0.2798 (4)	0.0368 (11)	
N6	0.9004 (5)	−0.2292 (4)	0.2531 (4)	0.0323 (10)	
C1	0.8958 (5)	0.1617 (5)	0.2043 (5)	0.0251 (11)	
C2	1.0526 (5)	0.2239 (5)	0.1442 (5)	0.0264 (11)	
C3	1.0755 (6)	0.3612 (5)	0.1080 (5)	0.0311 (12)	
H3	0.9909	0.4206	0.1266	0.037*	
C4	1.2251 (6)	0.4128 (5)	0.0433 (5)	0.0360 (13)	
H4	1.2443	0.5077	0.0165	0.043*	
C5	1.3438 (6)	0.3223 (5)	0.0196 (5)	0.0395 (14)	
H5	1.4465	0.3543	−0.0251	0.047*	
C6	1.3123 (6)	0.1852 (5)	0.0613 (5)	0.0355 (13)	
H6	1.3959	0.1235	0.0459	0.043*	
C7	0.6341 (5)	0.1688 (5)	0.2782 (5)	0.0268 (11)	
C8	0.4833 (5)	0.2407 (5)	0.3123 (5)	0.0284 (11)	
C9	0.3490 (5)	0.1626 (5)	0.3722 (5)	0.0324 (12)	
H9	0.3537	0.0668	0.3893	0.039*	
C10	0.2098 (6)	0.2279 (6)	0.4058 (5)	0.0405 (14)	
H10	0.1164	0.1774	0.4479	0.049*	
C11	0.2074 (6)	0.3662 (6)	0.3781 (6)	0.0446 (14)	
H11	0.1130	0.4128	0.4028	0.053*	
C12	0.3452 (6)	0.4377 (6)	0.3130 (6)	0.0419 (14)	
H12	0.3416	0.5345	0.2909	0.050*	
C13	0.7563 (5)	−0.0299 (5)	0.2721 (5)	0.0278 (11)	
C14	0.7581 (5)	−0.1748 (5)	0.2860 (5)	0.0272 (11)	
C15	0.6218 (6)	−0.2477 (5)	0.3296 (5)	0.0337 (12)	
H15	0.5226	−0.2055	0.3503	0.040*	
C16	0.6351 (6)	−0.3834 (6)	0.3417 (6)	0.0429 (14)	
H16	0.5445	−0.4373	0.3741	0.051*	
C17	0.7807 (6)	−0.4417 (6)	0.3066 (6)	0.0428 (14)	
H17	0.7919	−0.5348	0.3131	0.051*	
C18	0.9087 (6)	−0.3594 (5)	0.2619 (5)	0.0379 (13)	
H18	1.0086	−0.3977	0.2359	0.045*	

### Atomic displacement parameters (Å^2^)

**Table:**

	*U*^11^	*U*^22^	*U*^33^	*U*^12^	*U*^13^	*U*^23^
Mn1	0.0200 (4)	0.0324 (5)	0.0388 (5)	0.0006 (3)	−0.0039 (3)	−0.0120 (4)
Cl1	0.0270 (8)	0.0674 (11)	0.0416 (8)	−0.0058 (7)	−0.0071 (6)	−0.0086 (7)
Cl2	0.0349 (8)	0.0593 (10)	0.0476 (9)	0.0063 (7)	−0.0022 (6)	−0.0259 (7)
N1	0.017 (2)	0.028 (2)	0.036 (2)	−0.0024 (16)	−0.0035 (17)	−0.0133 (19)
N2	0.022 (2)	0.029 (2)	0.033 (2)	−0.0064 (17)	−0.0035 (17)	−0.0136 (19)
N3	0.018 (2)	0.028 (2)	0.037 (2)	−0.0030 (17)	−0.0009 (17)	−0.0132 (19)
N4	0.017 (2)	0.032 (2)	0.033 (2)	−0.0043 (17)	0.0016 (17)	−0.0104 (19)
N5	0.026 (2)	0.035 (3)	0.052 (3)	0.0091 (19)	−0.012 (2)	−0.020 (2)
N6	0.027 (2)	0.028 (2)	0.045 (3)	0.0023 (18)	−0.0091 (19)	−0.017 (2)
C1	0.020 (3)	0.019 (3)	0.032 (3)	−0.0023 (19)	−0.002 (2)	−0.007 (2)
C2	0.015 (2)	0.031 (3)	0.033 (3)	−0.001 (2)	−0.004 (2)	−0.012 (2)
C3	0.028 (3)	0.024 (3)	0.039 (3)	−0.004 (2)	−0.007 (2)	−0.008 (2)
C4	0.027 (3)	0.036 (3)	0.040 (3)	−0.013 (2)	−0.006 (2)	−0.006 (2)
C5	0.026 (3)	0.041 (4)	0.042 (3)	−0.009 (2)	−0.005 (2)	−0.007 (3)
C6	0.021 (3)	0.039 (3)	0.041 (3)	−0.002 (2)	0.001 (2)	−0.014 (3)
C7	0.021 (3)	0.029 (3)	0.030 (3)	−0.001 (2)	−0.005 (2)	−0.012 (2)
C8	0.024 (3)	0.030 (3)	0.030 (3)	0.000 (2)	−0.006 (2)	−0.010 (2)
C9	0.023 (3)	0.036 (3)	0.034 (3)	−0.001 (2)	−0.005 (2)	−0.009 (2)
C10	0.024 (3)	0.054 (4)	0.037 (3)	0.004 (3)	−0.005 (2)	−0.014 (3)
C11	0.032 (3)	0.059 (4)	0.044 (3)	0.014 (3)	−0.015 (3)	−0.022 (3)
C12	0.045 (4)	0.040 (3)	0.050 (4)	0.010 (3)	−0.020 (3)	−0.023 (3)
C13	0.018 (3)	0.033 (3)	0.030 (3)	−0.007 (2)	−0.002 (2)	−0.009 (2)
C14	0.024 (3)	0.026 (3)	0.033 (3)	−0.003 (2)	−0.006 (2)	−0.012 (2)
C15	0.026 (3)	0.035 (3)	0.040 (3)	−0.006 (2)	−0.003 (2)	−0.015 (3)
C16	0.042 (4)	0.036 (3)	0.053 (4)	−0.015 (3)	−0.004 (3)	−0.017 (3)
C17	0.045 (4)	0.032 (3)	0.052 (4)	−0.008 (3)	−0.005 (3)	−0.018 (3)
C18	0.041 (3)	0.027 (3)	0.052 (4)	0.006 (2)	−0.012 (3)	−0.022 (3)

### Geometric parameters (Å, °)

**Table:**

Mn1—N1	2.197 (4)	C4—H4	0.9500
Mn1—N4	2.303 (4)	C5—C6	1.376 (7)
Mn1—N6	2.324 (4)	C5—H5	0.9500
Mn1—Cl2	2.3345 (16)	C6—H6	0.9500
Mn1—Cl1	2.3494 (16)	C7—C8	1.490 (6)
N1—C1	1.329 (6)	C8—C9	1.395 (6)
N1—C13	1.339 (5)	C9—C10	1.375 (7)
N2—C1	1.335 (6)	C9—H9	0.9500
N2—C7	1.355 (5)	C10—C11	1.367 (7)
N3—C13	1.317 (6)	C10—H10	0.9500
N3—C7	1.345 (6)	C11—C12	1.390 (7)
N4—C6	1.343 (5)	C11—H11	0.9500
N4—C2	1.351 (6)	C12—H12	0.9500
N5—C8	1.336 (6)	C13—C14	1.474 (6)
N5—C12	1.339 (6)	C14—C15	1.386 (6)
N6—C18	1.332 (6)	C15—C16	1.378 (7)
N6—C14	1.342 (6)	C15—H15	0.9500
C1—C2	1.488 (6)	C16—C17	1.388 (7)
C2—C3	1.369 (6)	C16—H16	0.9500
C3—C4	1.398 (6)	C17—C18	1.380 (7)
C3—H3	0.9500	C17—H17	0.9500
C4—C5	1.375 (7)	C18—H18	0.9500
			
N1—Mn1—N4	70.05 (13)	N4—C6—H6	118.4
N1—Mn1—N6	70.68 (14)	C5—C6—H6	118.4
N4—Mn1—N6	137.65 (14)	N3—C7—N2	124.9 (4)
N1—Mn1—Cl2	139.00 (11)	N3—C7—C8	115.2 (4)
N4—Mn1—Cl2	104.03 (11)	N2—C7—C8	119.9 (4)
N6—Mn1—Cl2	95.27 (11)	N5—C8—C9	123.6 (5)
N1—Mn1—Cl1	108.53 (11)	N5—C8—C7	118.4 (4)
N4—Mn1—Cl1	103.39 (11)	C9—C8—C7	118.0 (4)
N6—Mn1—Cl1	103.49 (11)	C10—C9—C8	118.3 (5)
Cl2—Mn1—Cl1	112.22 (6)	C10—C9—H9	120.9
C1—N1—C13	116.3 (4)	C8—C9—H9	120.9
C1—N1—Mn1	122.6 (3)	C11—C10—C9	119.2 (5)
C13—N1—Mn1	121.1 (3)	C11—C10—H10	120.4
C1—N2—C7	113.9 (4)	C9—C10—H10	120.4
C13—N3—C7	115.7 (4)	C10—C11—C12	118.9 (5)
C6—N4—C2	117.2 (4)	C10—C11—H11	120.5
C6—N4—Mn1	124.6 (3)	C12—C11—H11	120.5
C2—N4—Mn1	117.9 (3)	N5—C12—C11	123.3 (5)
C8—N5—C12	116.7 (4)	N5—C12—H12	118.3
C18—N6—C14	117.5 (4)	C11—C12—H12	118.3
C18—N6—Mn1	125.3 (3)	N3—C13—N1	123.9 (5)
C14—N6—Mn1	116.8 (3)	N3—C13—C14	120.5 (4)
N1—C1—N2	125.0 (4)	N1—C13—C14	115.6 (4)
N1—C1—C2	114.3 (4)	N6—C14—C15	123.3 (5)
N2—C1—C2	120.8 (4)	N6—C14—C13	114.9 (4)
N4—C2—C3	123.0 (4)	C15—C14—C13	121.8 (4)
N4—C2—C1	114.0 (4)	C16—C15—C14	117.7 (5)
C3—C2—C1	122.9 (4)	C16—C15—H15	121.1
C2—C3—C4	119.1 (5)	C14—C15—H15	121.1
C2—C3—H3	120.5	C15—C16—C17	120.2 (5)
C4—C3—H3	120.5	C15—C16—H16	119.9
C5—C4—C3	118.2 (5)	C17—C16—H16	119.9
C5—C4—H4	120.9	C18—C17—C16	117.4 (5)
C3—C4—H4	120.9	C18—C17—H17	121.3
C4—C5—C6	119.4 (5)	C16—C17—H17	121.3
C4—C5—H5	120.3	N6—C18—C17	123.8 (5)
C6—C5—H5	120.3	N6—C18—H18	118.1
N4—C6—C5	123.1 (5)	C17—C18—H18	118.1
			
N4—Mn1—N1—C1	−8.9 (3)	C3—C4—C5—C6	−0.8 (8)
N6—Mn1—N1—C1	−172.7 (4)	C2—N4—C6—C5	−0.6 (7)
Cl2—Mn1—N1—C1	−97.5 (4)	Mn1—N4—C6—C5	−174.8 (4)
Cl1—Mn1—N1—C1	89.0 (4)	C4—C5—C6—N4	1.3 (8)
N4—Mn1—N1—C13	172.3 (4)	C13—N3—C7—N2	2.1 (7)
N6—Mn1—N1—C13	8.4 (3)	C13—N3—C7—C8	−178.6 (4)
Cl2—Mn1—N1—C13	83.7 (4)	C1—N2—C7—N3	−1.6 (7)
Cl1—Mn1—N1—C13	−89.8 (4)	C1—N2—C7—C8	179.0 (4)
N1—Mn1—N4—C6	−176.4 (4)	C12—N5—C8—C9	2.8 (7)
N6—Mn1—N4—C6	−153.5 (4)	C12—N5—C8—C7	−179.5 (4)
Cl2—Mn1—N4—C6	−39.0 (4)	N3—C7—C8—N5	−172.6 (4)
Cl1—Mn1—N4—C6	78.4 (4)	N2—C7—C8—N5	6.8 (7)
N1—Mn1—N4—C2	9.4 (3)	N3—C7—C8—C9	5.2 (6)
N6—Mn1—N4—C2	32.4 (4)	N2—C7—C8—C9	−175.4 (4)
Cl2—Mn1—N4—C2	146.9 (3)	N5—C8—C9—C10	−3.3 (7)
Cl1—Mn1—N4—C2	−95.7 (3)	C7—C8—C9—C10	179.0 (4)
N1—Mn1—N6—C18	178.9 (4)	C8—C9—C10—C11	0.8 (7)
N4—Mn1—N6—C18	156.1 (4)	C9—C10—C11—C12	1.8 (8)
Cl2—Mn1—N6—C18	38.5 (4)	C8—N5—C12—C11	0.1 (7)
Cl1—Mn1—N6—C18	−75.9 (4)	C10—C11—C12—N5	−2.4 (8)
N1—Mn1—N6—C14	−8.5 (3)	C7—N3—C13—N1	1.9 (7)
N4—Mn1—N6—C14	−31.4 (5)	C7—N3—C13—C14	−177.6 (4)
Cl2—Mn1—N6—C14	−148.9 (3)	C1—N1—C13—N3	−5.8 (7)
Cl1—Mn1—N6—C14	96.7 (3)	Mn1—N1—C13—N3	173.0 (4)
C13—N1—C1—N2	6.4 (7)	C1—N1—C13—C14	173.7 (4)
Mn1—N1—C1—N2	−172.5 (4)	Mn1—N1—C13—C14	−7.5 (5)
C13—N1—C1—C2	−173.8 (4)	C18—N6—C14—C15	0.8 (7)
Mn1—N1—C1—C2	7.3 (5)	Mn1—N6—C14—C15	−172.4 (4)
C7—N2—C1—N1	−2.8 (7)	C18—N6—C14—C13	−179.0 (4)
C7—N2—C1—C2	177.4 (4)	Mn1—N6—C14—C13	7.8 (5)
C6—N4—C2—C3	−0.7 (7)	N3—C13—C14—N6	178.8 (4)
Mn1—N4—C2—C3	173.9 (4)	N1—C13—C14—N6	−0.7 (6)
C6—N4—C2—C1	176.3 (4)	N3—C13—C14—C15	−1.0 (7)
Mn1—N4—C2—C1	−9.1 (5)	N1—C13—C14—C15	179.5 (4)
N1—C1—C2—N4	1.6 (6)	N6—C14—C15—C16	1.3 (8)
N2—C1—C2—N4	−178.5 (4)	C13—C14—C15—C16	−178.9 (4)
N1—C1—C2—C3	178.6 (4)	C14—C15—C16—C17	−2.1 (8)
N2—C1—C2—C3	−1.6 (7)	C15—C16—C17—C18	0.9 (8)
N4—C2—C3—C4	1.2 (7)	C14—N6—C18—C17	−2.1 (8)
C1—C2—C3—C4	−175.5 (4)	Mn1—N6—C18—C17	170.4 (4)
C2—C3—C4—C5	−0.4 (7)	C16—C17—C18—N6	1.2 (8)

### Hydrogen-bond geometry (Å, °)

**Table:**

*D*—H···*A*	*D*—H	H···*A*	*D*···*A*	*D*—H···*A*
C10—H10···Cl1^i^	0.95	2.77	3.594 (5)	145
C15—H15···Cl1^ii^	0.95	2.82	3.714 (5)	157

Symmetry codes: (i) −*x*+1, −*y*, −*z*+1; (ii) *x*−1, *y*, *z*.
